# Glenohumeral and scapulothoracic strength impairments exists in patients with subacromial impingement, but these are not reflected in the shoulder pain and disability index

**DOI:** 10.1186/s12891-017-1667-1

**Published:** 2017-07-17

**Authors:** M.B. Clausen, A. Witten, K. Holm, K.B. Christensen, M.L. Attrup, P. Hölmich, K. Thorborg

**Affiliations:** 1 Department of Physiotherapy and Occupational Therapy, Faculty of Health and Technology, Metropolitan University College, Sigurdsgade 26, DK-2200 Copenhagen, Denmark; 2 Sports Orthopedic Research Center - Copenhagen, Department of Orthopedic Surgery, Copenhagen University Hospital, Amager-Hvidovre, Denmark; 3 Department of Biostatistics, University of Copenhagen, Copenhagen, Denmark; 4 Physical Medicine and Rehabilitation Research-Copenhagen (PMR-C), Amager-Hvidovre Hospital, Copenhagen University Hospital, Copenhagen, Denmark

**Keywords:** Strength, Self report, Shoulder, Impairment, Deficit, Range of motion, Pain

## Abstract

**Background:**

Pain and loss of function are cardinal symptoms associated with Subacromial impingement syndrome (SIS), while the presence and magnitude of deficits in strength and range of motion (ROM) are largely undescribed in non-athletic patients with SIS. Moreover, the relevance of impairments in strength and ROM to patient-reported shoulder function is not well described, even though testing of strength is recommended in clinical guidelines.

The purpose of this study was, first, to investigate impairments in glenohumeral and scapulothoracic strength and in abduction and internal rotation ROM in patients with SIS. Secondly, to investigate the influence of these impairments on patient-reported shoulder function.

**Methods:**

Cross-sectional study based on a consecutive cohort of 157 patients referred to specialist examination and diagnosed with shoulder impingement (SIS) using predefined validated diagnostic criteria. Prior to specialist examination, questionnaires regarding shoulder function (Shoulder Pain And Disability Index, SPADI) demographics and kinesiophobia (TSK-11) were collected, and shoulder strength and ROM was measured by trained testers, with the patient reporting pain levels during testing and for the last week. Impairments in strength (abduction, external-rotation, (protraction and horizontal-extension) and ROM (abduction and internal rotation) were investigated in patients with unilateral shoulder pain, using one-sample t-tests. SPADI total score (SPADI) and SPADI function score (SPADI-F), were chosen as dependent variables in multiple regressions to investigate the influence of impairments on patient-reported shoulder function. Independent variables of interest were; strength in abduction and external rotation, abduction ROM, pain-during-tests, pain-last-week and kinesiophobia.

**Results:**

Significant impairments were found for all impairment tests, but most pronounced for glenohumeral strength and abduction ROM (29–33% deficits), and less for scapulothoracic strength and internal rotation ROM (8–18% deficits). Pain variables influenced SPADI and SPADI-F score to a high degree (R^2^ = 23.4–31.6%, *p* < 0.001), while strength and ROM did not.

**Conclusion:**

Substantial strength and ROM impairments were found in patients with SIS. Only pain significantly influenced patient-reported function, while impairments did not. As SPADI score does not reflect the substantial strength and ROM impairments in external rotation and abduction observed in patients with SIS, supplemental assessment of these impairments seems important.

**Electronic supplementary material:**

The online version of this article (doi:10.1186/s12891-017-1667-1) contains supplementary material, which is available to authorized users.

## Background

Subacromial impingement syndrome (SIS) is one of the musculoskeletal conditions that most frequently leads adults to contact a general practitioner [1, 2]. Subjective sensations of pain and loss of function are cardinal symptoms associated with SIS [3]. When monitoring such concepts, which are best known by the patient, the use of patient-reported outcomes is advisable [4], and the use of Patient-reported outcomes is also recommended in a range of clinical guidelines for the management of SIS [5–7]. In addition to this, assessment of strength and range of motion (ROM) is recommended in clinical guidelines [6, 7], although not consistently [5]. These recommendations are based on consensus decisions [6, 7], and most likely derive from the assumption that patients with SIS generally have strength and ROM deficits. However, the guidelines lack specificity, as there are no recommendations specifying in which directions of movement shoulder strength and ROM should be tested when assessing patients with SIS, and whether tests of both glenohumeral and scapulothoracic functions should be included [6, 7]. This lack of specificity is in line with the current available evidence. Accordingly, while levels of strength and ROM is reported in some clinical trials [8–12], the magnitude of deficits have only been quantified for rotation strength (24–37% deficit) [13, 14] and passive internal rotation ROM (~10% deficit) [15, 16] in a non-athletic SIS population. With the limited knowledge about the magnitude of strength and ROM deficits in patients with SIS being, especially regarding scapulothoracic function, no recommendations about relevance of shoulder strength testing in specific directions of movement can be made.

The recommendations that shoulder strength should be assessed as part of the management of patients with SIS [6, 7] could also be debated, as shoulder strength is found to be highly related to shoulder function [13], and hence would be reflected in the Patient-reported outcome score. This has, however, only been investigated in one previous study by MacDermid et al. [13].

However, these results only concerns external rotation strength and are based on a small convenience sample of 36 patients with SIS, thus limiting the external validity of these findings. In addition, no adjustment was made for covariates such as age, gender, pain and kinesiophobia; covariates, which are possible confounders, as they are likely associated with shoulder strength, and have previously been found to significantly influence patient-reported shoulder function [17, 18]. With regard to the relationship between scapulothoracic strength and patient-reported shoulder function, this has, to the best of our knowledge, not previously been investigated. Collectively, the above reveals a scarcity of literature investigating how strength is related to shoulder function in patients with SIS. Consequently, the degree to which glenohumeral and scapulothoracic strength impairments are reflected in patient-reported outcomes in patients with SIS, is not well understood. Therefore, further research investigating this, taking important covariates into account, is needed. Such knowledge will assist clinicians and researchers in determining the relevance of including objective strength testing. This is especially relevant as a substantial part of current SIS rehabilitation programs include glenohumeral and scapulothoracic strengthening exercises [8, 19]. In addition, an improved understanding of the impact that strength, ROM and pain impairments have on the patient’s experience of the severity of their shoulder disorder, will facilitate the discussion concerning the importance of addressing these impairments in the rehabilitation.

## Methods

### Aim

The aims of this study were: First, to describe and quantify possible strength and mobility deficits related to maximum isometric strength in shoulder abduction, external rotation, protraction, and horizontal extension, in active abduction ROM and in passive internal rotation ROM in patients with SIS, to elaborate on clinical recommendations regarding the choice of movement directions in which shoulder strength and mobility should be monitored. Secondly, to investigate the influence of strength and ROM impairments on patient-reported shoulder function, evaluated by the function score of the Shoulder Pain And Disability Index (SPADI-F score), in patients with SIS, when adjusted for important covariates, to understand the importance of supplemental assessment of strength and ROM impairments in these patients.

### Design

Cross-sectional consecutive cohort study.

### Settings and procedures

This study includes a consecutive cohort of 157 patients with SIS, all referred to an orthopedic shoulder specialist at Arthroscopic Center Amager, for examination of a shoulder problem, during a 3-month period (March to June 2014). During this period, all patients referred to examination of their shoulder problem at the department (e.g. by their general practitioner) received a letter containing information that an extended examination was offered. The extended examination included test of passive internal rotation ROM, active abduction ROM and external rotation strength, as these are reported low or impaired in patients with SIS [13, 15–17]. Maximum isometric strength in abduction was also tested, as evidence regarding the magnitude of deficits in abduction strength seems scarce, even though abduction strengthening exercises are included in the most novel rehabilitation intervention to patients with SIS [12, 19]. Tests of maximum isometric strength in horizontal extension and protraction in the shoulder were included to investigate scapulothoracic muscle function. All outcome assessments were conducted before the clinical examination performed by an orthopedic shoulder specialist, who was blinded to all test results. This study is part of a larger project, including all shoulder patients referred to the department during the 3-month period, and further data on this cohort will be reported in subsequent papers. Informed consent was received from all participants and the rights of the subjects were protected.

### Participants

**Fig. 1:**
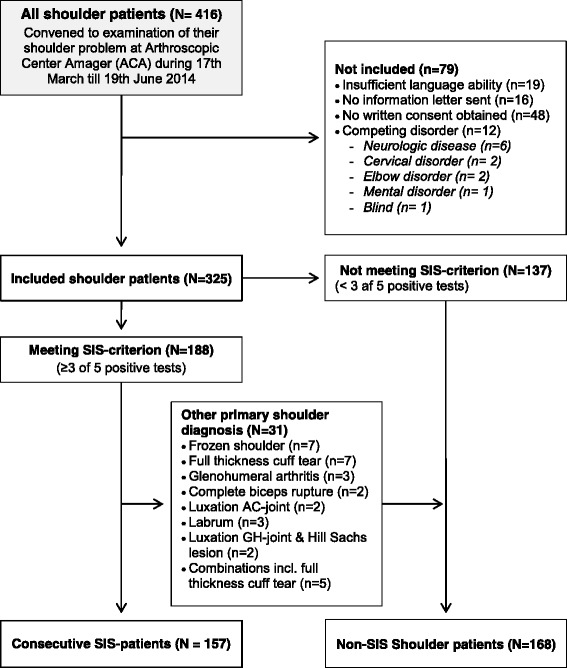
Flow-chart

Patients were considered eligible for inclusion in the consecutive cohort of shoulder patients based on the following inclusion criteria: Aged 18 years or more; referred to examination of a shoulder problem, sufficient Danish language ability; and no competing disorder affecting the shoulder function or the ability to answer patient-reported questionnaires was present (e.g. neurologic disease, cervical disorder, elbow disorder, mental disorder and blindness). Patients were further included as consecutive patients with SIS if meeting the predefined SIS-criterion shown by Michener et al. [20, 21] to have the highest diagnostic accuracy, where at least three positive of the five diagnostic tests for SIS should be present (Hawkins-Kennedy, Neer’s, pain-full arc, Resisted External Rotation and Jobe’s). The standard examination, including all diagnostic tests, was conducted by one of the orthopedic shoulder specialists at the department. The orthopedic shoulder specialists were all familiar with the five impingement tests, and in addition, the performance and interpretation of these tests were also discussed at a staff meeting prior to initiation of the study. Patients were excluded from the study if clinical and/or para-clinical (Ultrasound, MRI etc.) examination revealed a full thickness rotator cuff tear, luxation or sub-luxation of the glenohumeral or the acromioclavicular joint, frozen shoulder or osteoarthritis in the glenohumeral joint; or if a labrum lesion verified by para-clinical investigation was identified, based on the clinical judgement of the orthopedic surgeon shoulder specialist performing the examination. For further information on study flow, see Fig. 1.

### Outcome measures

Patient-reported shoulder function was measured using the Danish version of the Shoulder Pain And Disability Index (SPADI) [22]. SPADI is a shoulder-specific questionnaire consisting of 13 questions, each scored on an 11-point numeric rating scale [22]. SPADI consists of two domains measuring pain (SPADI-P, five questions) and function (SPADI-F, eight questions), respectively. Each domain is scored from 0 (best) to 100 (worst), and averaged into a total SPADI score. In a recent systematic review [23], the original English version of SPADI is highlighted as the Patient-reported outcome supported by the best evidence for patients with shoulder pain. SPADI is the only Danish shoulder specific Patient-reported outcome for which the psychometric properties have been evaluated, and the Danish SPADI is found to have good reliability (ICC 0.88) and known group validity in a population comparable to that included in the current study [22]. For the purpose of investigating the influence of impairments and pain on patient-reported function in this study, the SPADI-F score was, a priori, chosen as the primary dependent variable, as inclusion of the SPADI-P score is somewhat redundant with the pain intensity measures, with the consequent risk of multicollinearity in the regression models. However, similar analyses were also performed including SPADI score (the full questionnaire) as the dependent variable, as this is the score most often used in clinical and scientific settings.

Kinesiophobia was monitored with a Danish version of the shortened Tampa Scale of Kinesiophobia (TSK-11) [24]. Scores range from 11 to 44, with low scores indicating less kinesiophobia. The psychometric properties of the Danish version of Tampa Scale have not been investigated.

The following disease specific characteristics were collected; Duration (duration of current shoulder problem: 0–1 months/1–3 months/3–6 months/>6 months); Medication use (none/some days/most days/every day); Insurance status (ongoing or approved worker’s compensations claim, yes/no); Sick leave (on sick leave or unemployed due to shoulder problem, yes/no); Affected Side (dominant side/non-dominant side diagnosed with SIS); Age (years); and gender (male/female). The above mentioned disease specific characteristics; Duration, Medication use, Insurance status, Sick leave, Affected side, Age and Gender, were selected as relevant covariates to be included based on the existing literature [17, 25].

Clinical tests of shoulder impairments included reliable clinical measurements of ROM and maximum isometric strength of the affected shoulder. All tests were conducted by one from a team of trained testers, consisting of two physiotherapists, three bachelor students in physiotherapy and one medical student. All testers underwent thorough training over the duration of approximately 1 month, and did not perform any testing before approved by the primary investigator. Additionally, the unaffected shoulder was tested first for patients reporting no shoulder pain in the opposite shoulder, within the last 6 months.

Range of Motion (ROM) was measured in degrees using a digital inclinometer. Abduction ROM (Abd-ROM) was tested with the patient in a standing position, elevating the arm as high as possible in the frontal plane without lateral flexion of the spine. One familiarization trial was performed prior to the actual test. Internal rotation ROM (IR-ROM) was tested with the patient in side lying position on the shoulder being tested, the shoulder in 90 degrees of flexion, in a sleepers stretch position. One familiarization trial was performed, and the average of the following two tests was used as the test result. Both procedures have previously been described in the literature and are reported to have a high inter-tester reliability with ICC ≥ 0.95, minimal detectable change (MDC_90_) of 4° for abduction ROM and MDC_95_ = 6° for internal rotation ROM [15, 26], but the inter tester reliability of ROM testing was not investigated as part of the current study.

Four tests of maximum isometric peak torque were performed using a hand-held dynamometer; in abduction (Abd-Strength), external rotation (ER-Strength), horizontal extension (HE-Strength) and protraction (Pro-Strength). All maximum isometric testing procedures were developed as part of this project, and the full testing procedures are described in Additional file 1. ER-strength and Abd-strength was tested with the subject in seated position close to a wall, with the shoulder in neutral position, and the elbow flexed to 90 degrees or fully extended, respectively. The wall was used as external resistance to the isometric contraction performed by the subject. HE-strength was tested with the subject in prone position with the shoulder in 90 degrees of abduction and 0 degrees of horizontal extension and the forearm aligned horizontally. External resistance to the isometric contraction was applied by the assessor. Pro-Strength was tested with the subject seated with the back supported by a vertical bench in 90 degrees position, the shoulder and elbow in 90 degrees of flexion. A vertical board in front of the subject was used as external resistance. Tests of maximum isometric peak torque were measured in Newton-meter (Newton for Pro-Strength, as no lever is measured for that test) and standardised to body weight (Nm/kg or N/kg). For procedures regarding the measurement of lever arm, please see Additional file 1. As part of this project, the inter-tester reliability of the tests was investigated. Inter-tester reliability was high for Abd-strength, ER-Strength and Pro-Strength (ICC_2,1_ > 0.8), and acceptable for HE-strength (ICC_2,1_ = 0.79 with 95% CI 0.45 to 0.91). For further details on the reliability, please see Additional file 1.

Pain intensity was monitored using the validated 11-point numeric rating scale (0 = no pain, 10 = worst imaginable pain) [27]. Pain-last-week was calculated as the average of least pain and average pain last week, a composite measure demonstrating high reliability [27]. Pain-during-testing (0–10) (e.g. pAbd-ROM, pAbd-strength etc.) was recorded after each impairment test. A similar measure of pain during testing is found sufficiently reliable for tests of hip disorders [28].

### Data analysis

Descriptive statistics with means and standard deviations (SD) were applied for continuous variables, and numbers (percentages) for dichotomised variables.

Relative deficits in Abd-ROM, IR-ROM, Abd-Strength, ER-Strength, HE-Strength and Pro-Strength were calculated as the difference between sides as a percentage of the unaffected side, in the subgroup of patients reporting no pain in the contralateral shoulder within the last 6 months. A one sample t-test was applied in order to test if relative deficits were significant, with a significance level of 0.05.

The correlation between each of the dependent variables (SPADI and SPADI-F score, respectively) and each of the impairment measures; Abd-Strength, ER-Strength, Abd-ROM, the pain variables pAbd-Strength, pER-Strength, pAbd-ROM, pain-last-week and Kinesiophobia, was investigated using Pearson correlations. The variables IR-ROM, Pro-Strength and HE-Strength were not included in the correlation and regression analyses because we experienced that a substantial part of the patients (8 to 22, see Table 2), was not able to perform the test. This was a consequence of the applied testing procedures which required the patient to be side-lying on the affected shoulder (IR-ROM) or have their arm in 90° of flexion or abduction (Pro-Strength and HE-Strength, respectively).

For all included independent variables, separate hierarchical regression analyses were performed, investigating the influence of each variable on SPADI and SPADI-F score, respectively, as expressed by the adjusted R^2^–change (∆R^2^-adj.). Covariates were included as the first step in the regression model in order to obtain covariate adjusted ∆R^2^-adj. Estimates for each independent variable. The covariates Affected Side, Age and Gender were, a priori, chosen to be included in all covariate adjusted analyses. The Disease specific characteristics Duration, Medication use, Insurance status and Sick leave were only included as covariates if they significantly influenced SPADI-F score, (*p* < 0.05 for ∆R^2^-adj.), when adjusted for the a priori selected covariates. Furthermore, in the final stepwise multiple regressions, the covariates and independent variables were entered in four steps: step 1) Covariates; step 2) pain module (pAbd-Strength, pER-Strength and pAbd-ROM, and Pain-last-week); step 3) strength and ROM impairment module (Abd-Strength, ER-Strength, Abd-ROM); and step 4) Kinesiophobia module (TSK-11). The pain module was added first, to allow the results regarding the impairment and kinesiophobia modules to be adjusted for all pain variables. All assumptions for hierarchical regression were tested for all regression models. In case correlation between independent variables >0.7 was present, the variable presenting the highest *p*-value in the separate hierarchical regression analyses was excluded from the model. For all variables included in regression analyses, the issue of missing data was addressed using multiple imputation. This was done using the full conditional specification (FCS; van Buuren, 2007) as implemented in SPSS v.22. Ten imputations were used. ∆R^2^-adj.values were considered as small (≥1%), medium (≥9%) or large (≥25%), corresponding to the cut-points for R-values of 0.1, 0.3 and 0.5, respectively [29]. All analyses were conducted using IBM SPSS v22. A significance level of 0.05 was applied.

## Results

The 157 patients with SIS (56% females) included in this study were an average of 54 years (±13). Their mean SPADI and SPADI-F scores were 57 (±20) and 49 (±22), respectively and 81% reported that their current shoulder symptoms had at least lasted 3 months. For further descriptive statistics on the study sample, see Table 1. In the subgroup of patients reporting no pain in the contralateral shoulder within the last 6 months (*n* = 87), significant relative deficits were found for all investigated strength and ROM measures (*p* < 0.05), though most pronounced in Abd-Strength (−29.3%), ER-Strength (−32.8%) and Abd-ROM (−29.5%), see Table 2.

**Table 1:** Patients demographic characteristics

	N=	
Age *mean ± SD*	157	54 *± 13*
SPADI total *± SD*	156	57 *± 20*
SPADI-F *± SD*	156	49 *± 22*
Kinesiophobia (TSK-11) *± SD*	148	28 *± 5*
Gender female, *n (%)*	88/157	(56.1%)
Dominant side affected *n(%)*	82/151	(54.3%)
Pain opposite shoulder within 6 months *n(%)*	64/151	(42.4%)
Duration of disorder, *n (%)*		
*0–1 month*	2/154	(1.3%)
*1–3 months*	27/154	(17.5%)
*3–6 months*	33/154	(21.4%)
*> 6 months*	92/154	(59.7%)
Sick Leave^a^ (% on sick leave)	14/152	(9.2%)
Insurance^b^ (% yes)	10/152	(6.6%)
Medication Use^c^		
*None*	41/156	(26.3%)
*Some days*	49/156	(31.4%)
*Most days*	30/156	(19.2%)
*Every day*	36/156	(23.1%)

^a^Patients on sick leave or part time because of shoulder problem

^b^Insurance: yes = ongoing or accepted claim, no = no insurance claim

^c^Highest frequency of medication (over the counter or prescribed)

**Table 2:** Relative impairments in symptomatic shoulder compared to opposite shoulder in patients with SIS reporting no pain in the opposite shoulder within the last 6 months (*n* = 87)

	Number^a^	Asympt. shoulder	Sympt. shoulder	Relative Deficit^b^ (95%CI)		*p=*
Abd-ROM*, ° ± SD*	77	164° ±14	116° ±42	29.5%	(23.9–35.1%)	<.0001
IR-ROM*, ° ± SD*	69	141° ±11	124° ±15	11.9%	(9.2–14.6%)	<.0001
Abd-Strength*, Nm ± SD*	72	53.1 Nm ±33.0	37.6 Nm ±28.9	29.3%	(23.7–34.9%)	<.0001
ER-Strength*, Nm ± SD*	72	19.9 Nm ±8.3	13.9 Nm ±9.9	32.8%	(26.4.-39.2%)	<.0001
HE-Strength*, Nm ± SD*	43	25.6 Nm ±15.6	21.2 Nm ±16.3	18.0%	(9.4–26.5%)	.0001
Pro-Strength*, N ± SD*	49	234.0 N ± 131.5	204.9 N ± 121.4	8.4%	(0.7–16.0%)	.032

^a^For all tests, some data are missing because the patients had difficulties performing the tests or due to insufficient time to completion of the tests. The number of missing data due to difficulties is: Abd-ROM, 2 missing; IR-ROM, 8 missing; Abd-Strength, 3 missing; ER-Strength, 3 missing; HE-Strength, 22 missing; Pro-Strength, 13 missing. The number of missing data due to time is: Abd-ROM, 8 missing; IR-ROM, 10 missing; Abd-Strength, 12 missing; ER-Strength, 12 missing; HE-Strength, 22 missing; Pro-Strength, 25 missing

^b^Calculated as the difference divided by the test result in the asymptomatic shoulder

Results from the Pearson correlation analyses, showing the correlation between each independent variable and the SPADI and SPADI-F scores, respectively, are presented in Table 3. In general, Pain-last-week, pAbd-ROM and pER-strength were the variable most correlated to SPADI and SPADI-F scores (*R* = 0.42 to 0.64, *p* < .001), while the strength variables were not at all reflected in SPADI score (*R* = −0.18, *p* > 0.05) and to a small degree in SPADI-F score (*R* = 0.22, *p* < .05).

**Table 3:** Pearson correlations and separate hierarchical regression analyses showing the correlation between each independent variable and the dependent variables (SPADI and SPADI-F) as well as the variance in SPADI and SPADI-F score explained by each independent variable (*n* = 156)

Variables	SPADI		SPADI-F	
	Pearson’s *R*	∆R^2^-adj (adjusted^a^)	Pearson’s *R*	∆R^2^-adj (adjusted^a^)
Abd-ROM	−0.32***	2.0%*	−0.36***	3.4%*
Abd-Strength	−0.18	0.0%	−0.22*	0.2%
ER-Strength	−0.18	−0.1%	−0.22*	0.2%
pAbd-ROM	0.54****	18.3%****	0.48****	13.7%****
pAbd-Strength	0.28**	4.1%*	0.28**	5.1%**
pER-Strength	0.45***	10.2%***	0.42***	9.4%***
Pain-last-week	0.64****	25.0%****	0.55****	17.3%****
Tampa scale (TSK-11)	0.31***	4.5%**	0.24**	2.6%*

^*^ *p* < 0.05, ^**^ *p* < 0.01, ^***^ *p* < 0.001, ^****^ *p* < 0.0001

^a^adjusted for Age, Gender, Affected Side, Sick Leave and Medication Use

In all adjusted regression analyses, the covariates Age, Gender, Affected Side, Sick Leave and Medication Use were included as covariates, and these alone explained a total of 23.4% of the variance in SPADI-F score (*p* < 0.0001) and 23.6% of the variance in SPADI score (*p* < 0.0001). Duration and Insurance status was not included as covariates as these variables did not significantly influence SPADI-F score, (*p* > 0.05 for ∆R^2^-adj.), when adjusted for the a priori selected covariates. In the separate hierarchical regression analyses, investigating the influence of each independent variable on SPADI and SPADI-F score, ∆R^2^-adj.values were medium to high for Pain-last-week (17.3–25.0, *p* < .0001), medium for pER-Strength (9.4–10.2%, *p* < .001) and pAbd-ROM (13.7–18.3%, *p* < .0001), but small for Abd-ROM (2.0–3.4%, *p* < .05), pAbd-Strength (4.1–5.1%, *p* < .05) and Kinesiophobia (2.6–4.5%, *p* < .05). Abd-strength (*p* > .44) and ER-strength (*p* > .38) did not influence SPADI or SPADI-F score, see Table 3. Post-hoc separate hierarchical regression analyses with IR-ROM, Pro-Strength and HE-Strength as independent variables, respectively, showed that none of these significantly influenced SPADI or SPADI-F score, *p* < 0.05 (data not shown). In the stepwise multiple regression, only the pain module (step 2) influenced the SPADI and SPADI-F score, when adjusted for covariates (∆R^2^-adj. 31.6 and 23.4%, respectively, *p* < 0.001), and the final model explained a total of 47% of the variance in SPADI-F score and 55.5% of the variance in SPADI score, see Table 4.

**Table 4:** Final hierarchical regression analyses showing the additional variance in SPADI and SPADI-F score explained by each module included in the model

Modules of variables included in the model	SPADI		SPADI-F	
	R^2^-adj	∆R^2^-adj^e^	R^2^-adj.	∆R^2^-adj^e^
Step 1: Covariates^a^	23.6%	23.6%****	23.4%	23.4%****
Step 2: Pain module^b^	55.2%	31.6%****	46.8%	23.4%****
Step 3: Impairment module^c^	55.0%	−0.2%	47.2%	0.4%
Step 4: Kinesiophobia^d^	55.5%	0.5%	47.1%	−0.1%

^*^ *p* < 0.05, ^**^ *p* < 0.01, ^***^ *p* < 0.001, ^****^ *p* < 0.0001

^a^Age, Gender, Affected Side, Sick Leave, Medication Use

^b^pAbd-ROM, pAbd-Strength, pER-Strength, Pain-last-week

^c^Abd-ROM and ER-Strength. Abd-Strength was not included due to a risk of multicollinearity, as Abd-Strength and ER-Strength showed a correlation >0.7, when testing assumptions for the regression model

^d^Tampa scale of kinesiophobia (TSK-11)

^e^Additional variance explain by each step of the hierarchical regression model

## Discussion

In this study of 157 consecutive patients with SIS, clinical hand-held dynamometer tests of isometric strength revealed significant strength deficits in shoulder abduction (29%), external rotation (33%), horizontal extension (18%) and protraction (8%), in the subgroup (*n* = 87) of patients reporting no pain in the opposite shoulder. Significant ROM deficits in abduction (30%) and internal rotation (12%) were also identified in this group. Conversely, the pain variables significantly influenced patient-reported shoulder function (∆R^2^-adj. 23.4–31.6%,), while shoulder strength and range of motion had none or minimal influence (*n* = 156).

To the best of our knowledge, the deficits in scapulothoracic strength have not previously been quantified in non-athletic patients with SIS, hindering any making of inferences about whether glenohumeral or scapulothoracic deficits are most pronounced in these patients. However, as we found more pronounced strength deficits in abduction and external rotation of 29–33% compared to 8–18% in horizontal extension and protraction strength, it seems that glenohumeral muscle strength deficits are more pronounced than those of the scapulothoracic joint. Although strength deficits in abduction, horizontal extension and protraction strength have not previously been quantified in non-athletic patients with SIS, the presence of such deficits is supported by one previous study by Celik et al. [30], who reported that significant strength deficits exists in the same movement directions. Importantly, however, Celik et al. [30] did not report the magnitude of these deficits. The pronounced deficits in external rotation isometric strength identified in this study is also in accordance with the deficits in external rotation strength (11–37%) that have been reported in non-athletic patients with SIS when compared to healthy subjects [13, 14, 31]. Importantly, however, previous studies investigating this did not include a population based sample of patients with SIS, limiting the external validity of those results. In general, the presence of deficits in glenohumeral strength identified in this study may not be surprising, as manually assessed muscle weakness is part of the diagnostic criterion described by Michener et al. [21], which were applied in this study. However, the specific manual muscle tests used for diagnosis do not provide any quantification of the muscle weakness, why the dynamometer and inclinometer data presented in this study adds valuable information to elucidate the magnitude of these deficits. In addition, the findings from this study, that deficit in shoulder strength seems more pronounced in the glenohumeral joint than in the scapulothoracic joint, suggests that especially assessment of glenohumeral strength is relevant when monitoring patients with SIS.

We found that none of the investigated glenohumeral shoulder strength measures significantly influenced the patient-reported function in a population of non-operated patients with SIS. This is in contrast to one previous study reporting that isometric external rotation strength to a high degree influenced SPADI score (R^2^ = 36%), also in non-operated patients with SIS [13]. A limitation in that previous study is that no adjustments were made for important covariates such as Age, Gender, Affected Side, Sick Leave and Medication Use; covariates which in this study were found to significantly influence the SPADI-F score (∆R^2^-adj. 23.4%), and therefore possible confounders. Furthermore, the external validity of the results presented by MacDermid et al. [13] is limited, due to a small and non-representative sample of patients, diagnosed with SIS or rotator cuff tendinitis using no specific criteria, increasing the risk that their results represents a spurious finding. In contrast, the results from the present study are based on a large sample of consecutive patients with SIS from a large outpatient hospital clinic. Therefore, given the results from our study, we do not find it likely that maximal glenohumeral shoulder strength influences SPADI-scores to a very high degree in non-athletic patients with SIS. In addition, in Post Hoc analyses, we found that scapulothoracic strength also was not reflected in SPADI or SPADI-F score. The reason for this lack of relationship between self-reported function and objective measures of shoulder strength cannot be explained by the findings of the current study, but one suggestion could be that the patients merely conforms to their functional capacity, and therefore do not experience limited strength as a functional deficit.

Despite our finding, that glenohumeral shoulder strength does not seem to influence SPADI-scores, the significant deficits in shoulder strength, especially in the glenohumeral joint (29–33% deficit), suggests that strengthening exercises are an important part of the rehabilitation of patients with SIS. Furthermore, the apparent lack of relationship between shoulder strength and self-reported function does not necessarily diminish the importance of targeting shoulder strength impairments in the rehabilitation of these patients. Accordingly, the use of strengthening exercises in the rehabilitation of patients with SIS, is, to some degree, supported by Lombardi et al. [11], where progressive resistance training alone, was found superior to waiting-list control for improving self-reported function and pain, even though no significant differences in shoulder peak torque change was found between the two groups. In line with this, most novel rehabilitation programs for patients with SIS [8, 19, 32] contain both strengthening exercises aimed at the glenohumeral and the scapulothoracic joint, respectively, though none is emphasised more than the other. However, to further improve the rehabilitation of patients with SIS, research investigating the importance of targeting the specific glenohumeral and scapulothoracic strength deficits, respectively, is needed.

All pain variables included in the regression analyses significantly reflected SPADI and SPADI-F score, and especially Pain-last-week was reflected in SPADI-F and SPADI score to a high degree (Adjusted ∆R^2^-adj. 17.3 and 25.0%, respectively, *p* < .0001) (see Table 3). This is only slightly more than the 13–16% previously reported [17, 33], and supports the current assumption [17, 33] that pain is, to a significant degree, reflected in both SPADI and SPADI-F score.

In addition, we also included the variables defined as pain during tests; variables which have not previously been investigated in a similar context. Interestingly, we found that pain during Abd-ROM and ER-strength testing were reflected in SPADI score to a higher degree than pain during Abd-strength testing. This could indicate that pain during test of abduction ROM and external rotation strength is more representative for the functional status of these patients than is pain during test of abduction strength and thus could be an important and quick indication of individual progress in a busy clinical setting, where questionnaire information cannot be obtained at every visit.

While the relative deficits in Abd-ROM have not previously been quantified in a comparable population, the levels of Abd-ROM (116°) in the affected side, found in our, study is in line with the 124–134° previously reported by Engebretsen et al. [17] in a comparable population. As expected, the impact of Abd-ROM on SPADI and SPADI-F score was small in our study, though it is slightly higher than previously reported [17]. Collectively, ours and previously published results support the recommendation that test of ROM should be included in the management of patients with SIS [6, 7], as active abduction ROM is only reflected to a small degree in SPADI and SPADI-F scores. Furthermore, in line with the literature [18], we found that kinesiophobia had a significant but small influence on SPADI-F score in the separate hierarchical regression analyses. However, this association vanished when adjusted for all pain and impairment variables, indicating that kinesiophobia is not related to patient-reported function, aside from the inherent close relationship to pain variables.

An important strength of the current study is the application of consecutive sampling of patients, which strengthens the external validity of the study findings. It should be noted, however, that only patients referred to further examination of their shoulder disorder were included in his study, and the findings therefore cannot necessarily be generalized to other populations of patients with SIS. An additional strength of this study is the application of pre-defined clinical criteria for the diagnosis of SIS, which improves the reproducibility of the study and the possibility for future replication of study findings. In this study, the relative deficits were calculated as an index between the affected and the non-affected side, and it could be argued that the calculation of a group level symmetry index is not optimal if important side-to-side differences are expected between the dominant and non-dominant side and more patients are affected in either the dominant or non-dominant side. However, we do not believe this is a problem in our study, as approximately half of the patients were affected in the dominant side (51% for relative deficit in Abd-ROM), and because little or no differences in external rotation and abduction strength between dominant or non-dominant side are observed in non-athletic populations [34–36], such as the population included in this study. However, one important limitation should be considered when strength is measured as the force output in a given movement direction, which is the case in this study, as this does not allow for quantification of the contributions from each muscle or muscle group. Furthermore, there is a risk of type I error in the current study due to the utilization of multiple imputation to handle missing data and the high number of statistical tests performed. However, results from Post Hoc multiple hierarchical regression analyses, using the raw data set instead of imputed datasets, did not reveal any results altering the conclusions from the main analyses. Furthermore, it should be noted that adjusting for this, using a Bonferroni correction (data not shown), would not have affected any of the conclusions drawn in the current study, except the presence of significant deficits in protraction strength.

## Conclusion

Pronounced deficits in glenohumeral strength and in active abduction ROM was found in patients with SIS. The SPADI and SPADI-F scores mainly reflect pain level and not the substantial strength and ROM impairments observed in Patients with SIS. Therefore, supplemental assessment of glenohumeral strength and abduction ROM seems important, and is further emphasized by the magnitude of the deficits as was found in this study.

## Additional file

Additional file 1: Description and inter-tester reliability of four tests of maximum isometric shoulder strength. Additional file containing the description and inter-tester reliability of the four tests of maximum isometric shoulder strength applied in the study. (DOCX 911 kb)

## Abbreviations

Abd-ROM: Abduction range of motion
Abd-Strength: Maximum isometric strength in abduction
ER-Strength: Maximum isometric strength in external rotation
HE-Strength: Maximum isometric strength in horizontal extension
IR-ROM: Internal rotation range of motion
pAbd-ROM: Pain during testing of abduction range of motion
pAbd-strength: Pain during testing of maximum isometric strength in abduction
pER-Strength: Pain during testing of maximum isometric strength in external rotation
Pro-Strength: Maximum isometric strength in protraction
ROM: Range of motion
SIS: Subacromial impingement syndrome
SPADI: Shoulder pain and disability index
SPADI-F: Shoulder pain and disability index, function subscale
TSK-11: Tampa scale of kinesiophobia, 11 item version
