# Supplementary material for: Glenohumeral and scapulothoracic strength impairments exists in patients with subacromial impingement, but these are not reflected in the shoulder pain and disability index
Source: BMC Musculoskelet Disord. 2017 Jul 17;18:302. doi: 10.1186/s12891-017-1667-1 (PMC5513121; doi:10.1186/s12891-017-1667-1)
Supplement: Additional file 1: — Description and inter-tester reliability of four tests of maximum isometric shoulder strength. Additional file containing the description and inter-tester reliability of the four tests of maximum isometric shoulder strength applied in the study. (DOCX 911 kb) [file 12891_2017_1667_MOESM1_ESM.docx]

## Methods

The inter-tester reliability of tests of external rotation, abduction, horizontal extension and protraction maximum isometric force was investigated in twenty-five asymptomatic adults (mean age 40 years ±8), 9 female and 16 males. Participants were tested by two trained assessors with at least 10 min rest between test sessions. Order of examiners and order of tests were randomized for all participants, using an even distribution of all possible combinations of testing order and order of examiner. The same test order was used for both examiners.

**
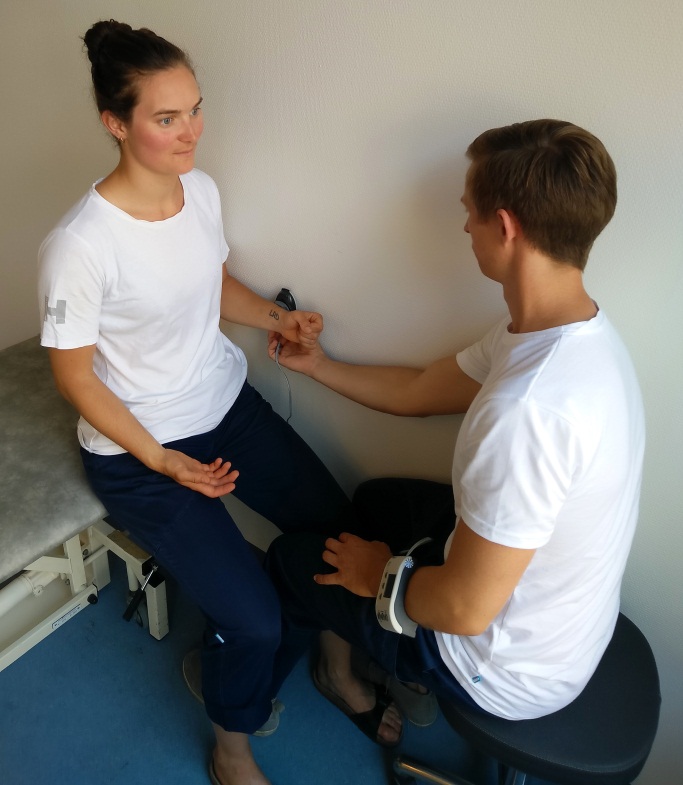
External rotation strength**The subject is seated on an examination table with adjustable height, with the knees and hips in 70 degrees of flexion, and both feet resting on the ground. The tested shoulder is in neutral position with 2-3 cm between the medial epicondyle of the humerus and the ribcage, and the elbow is in 90 degrees of flexion. The subject is seated close to the wall, allowing the dynamometer to be held between the wall and the distal aspect of the forearm. The JTECH Medical Commander Muscle tester Hand Held Dynamometer is placed dorsally on the forearm, with the distal aspect of the dynamometer’s sensor aligned just proximal to the radiocarpal joint. The subject is instructed to “sit up straight, shoulders back” and then asked to perform a 5 s isometric maximum voluntary contraction (MVC) against the dynamometer in a rotation movement, while the examiner is giving the standardized command "Go ahead-rotate-rotate-rotate-rotate and thank you". The examiner is positioned in front of the subject, to ensure force is only generated through external shoulder rotation, avoiding abduction and adduction in the shoulder. The test is administered two times, using the highest value as the test result. Each trial is followed by a 30 s rest period. The lever length is measured from the center of the dynamometer’s sensor to the lateral epicondyle of the humerus.

**Abduction strength**

The subject is seated on an examination table with adjustable height, with the knees and hips in 70 degrees of flexion, and both feet resting on the ground. The tested shoulder is in neutral position with 2-3 cm between the medial epicondyle of the humerus and the ribcage, and the elbow is in 0 degrees of flexion. The subject is seated close to the wall, allowing the dynamometer to be held between the wall and the distal aspect of the forearm. The JTECH Medical Commander Muscle tester Hand Held Dynamometer is placed dorsally on the forearm, with the distal aspect of the dynamometer’s sensor aligned just proximal to the radiocarpal joint. The subject is instructed to “sit up straight, shoulders back, straighten the arm” and then asked to perform a 5 s isometric maximum voluntary contraction (MVC) against the dynamometer in an abduction movement, while the examiner is giving the standardized command "Go ahead-push-push-push-push and thank you". The examiner is positioned in front of the subject, to ensure force is only generated through abduction, avoiding elevation of the shoulder and lateral flexion of the columna. The test is administered two times, using the highest value as the test result. Each trial is followed by a 30 s rest period. The lever length is measured from the center of the dynamometer’s sensor to the lateral aspect of the acromion.


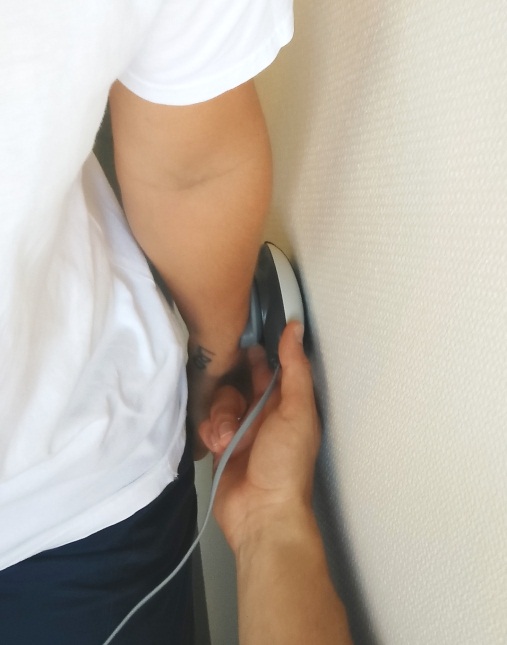

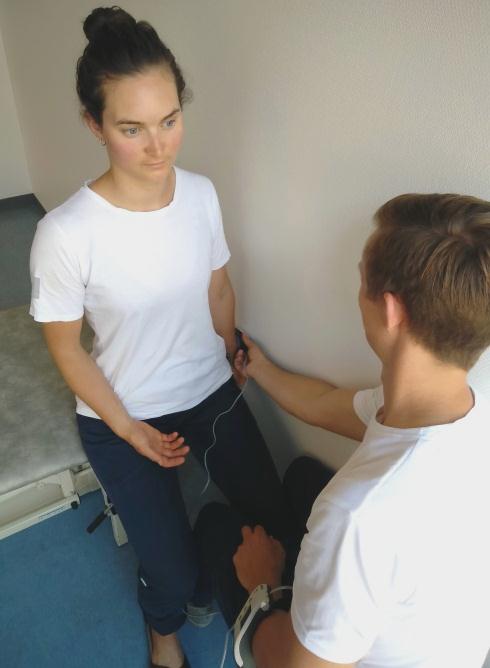


**Horizontal extension strength**

The subject is positioned in prone position on an examination table with adjustable height, with both shoulders in 90 degrees of abduction and the forearms hanging from the examination table. The tested shoulder is extended to 0 degrees, while the opposite arm holds on to the framework of the examination table. The feet are resting on a pillow. The height of the examination table is adjusted, to allow the examiner to use his upper body weight to keep the dynamometer in position, when force is exerted by the subject. The JTECH Medical Commander Muscle tester Hand Held Dynamometer is placed with the distal aspect of the dynamometer’s sensor aligned just proximal to the olecranon. The test arm is positioned in the correct position by the examiner; the subject is instructed to “keep this position” and then asked to perform a 5 s isometric maximum voluntary contraction (MVC) against the dynamometer in an abduction movement, while the examiner is giving the standardized command "Go ahead-push-push-push-push and thank you". The examiner applies isometric resistance, while the subject exerts maximum effort against the dynamometer in terms of a “make test”. The examiner ensures force is only generated through horizontal extension, without the use of rotation in the columna. The subject is not allowed to lift the feet or forehead. The test is administered two times, using the highest value as the test result. Each trial is followed by a 30 s rest period. The lever length is measured from the center of the dynamometer’s sensor to the lateral aspect of the acromion.

**
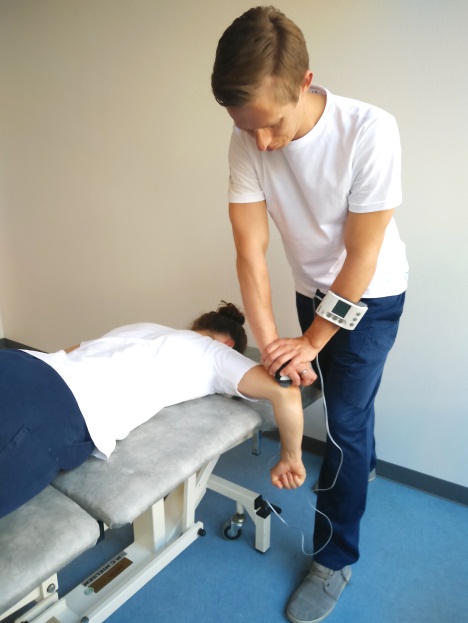

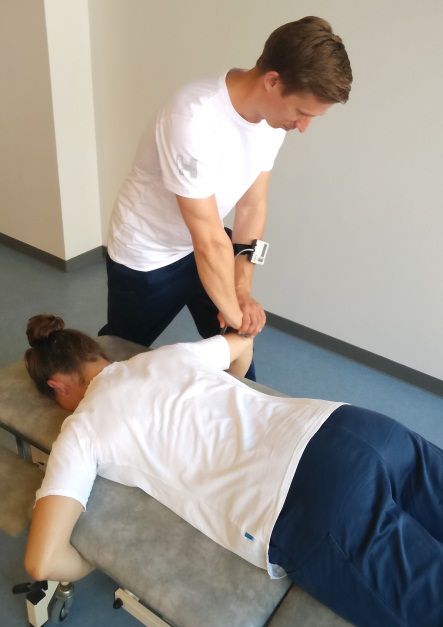
**

**Protraction strength**

The subject is seated, with both feet resting on the ground, and the back supported by a vertical bench in 90 degrees position. The tested shoulder and elbow is in 90 degrees of flexion. The subject is seated close to an adjustable vertical board, allowing the dynamometer to be held between the board and the forearm. The JTECH Medical Commander Muscle tester Hand Held Dynamometer is placed dorsally, with the distal aspect of the dynamometer’s sensor aligned just proximal to the olecranon. The board is adjusted, so the shoulder is protracted half of its range, when supporting the dynamometer. The subject exerts maximum effort against the dynamometer. The examiner ensures force is only generated through protraction, without the use of rotation or flexion in columna. The test is administered two times, using the highest value as the test result. Each trial is followed by a 30 s rest period


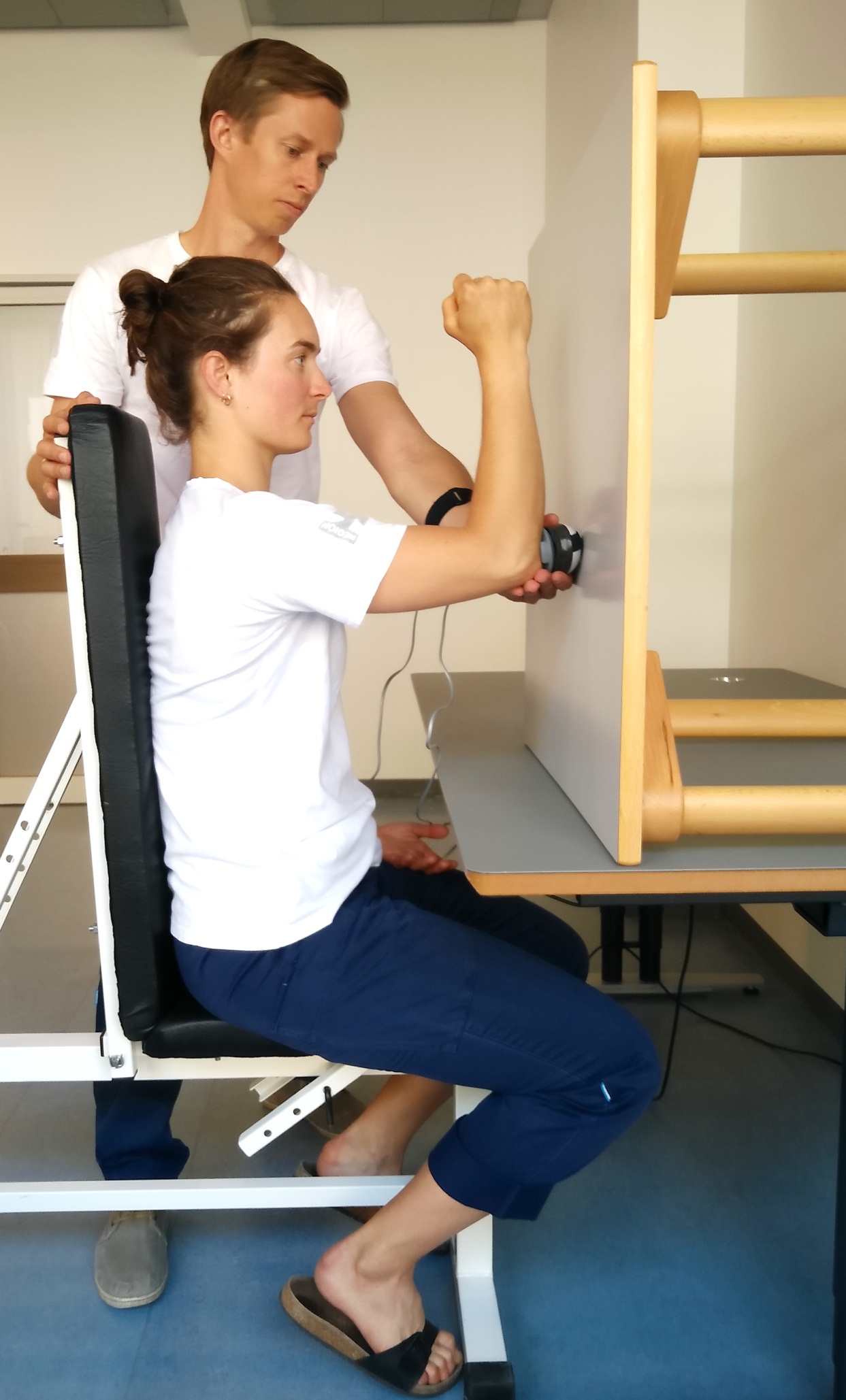

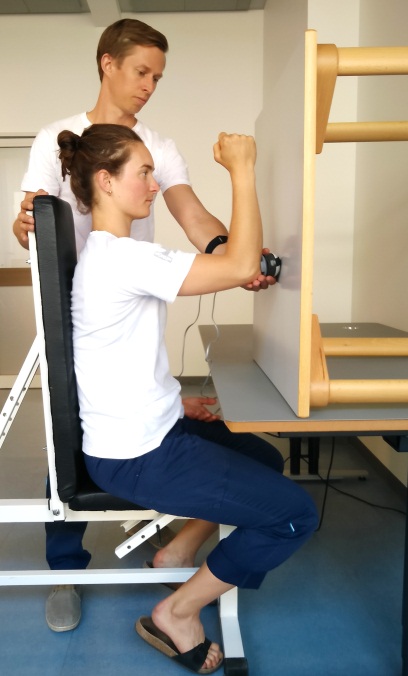


**Statistics**

Results are presented in table e1. The average for each test is presented as mean ±1 standard deviation (SD). Relative reliability and corresponding 95% confidence interval were calculated for all tests using intra-class correlation coefficient two-way mixed model - absolute agreement (ICC_2,1_), thereby taking any bias between testers into account. Absolute reliability was calculated as Standard Error of Measurement ($SEM=SD\sqrt{1-ICC}$, where SD is the standard deviation of all test results), SEM$\%=\frac{SEM}{mean of test 1 and 2}*100\%$ and Minimal Detectable Change ($MDC=SEM*1.96*\sqrt{2}$)

## Results

Results are presented in table e1. Tests of external rotation (ICC_2,1_=0.91, SEM=11.7N), abduction (ICC_2,1_=0.93, SEM=11.2N) and protraction (ICC_2,1_=0.92, SEM=78.2N) demonstrated good relative inter-tester reliability, with 95% confidence intervals of ICC_2,1_ not extending below 0.7. Test of horizontal extension (ICC_2,1_=0.79, SEM=53.4N) demonstrated acceptable to low inter-tester reliability, with an ICC_2,1_ estimate of 0.79, but a 95% confidence interval extending below 0.7.

| **Table e1** Inter-tester reliabilitet for four tests of maximum isometric shoulder strength (N=25) | | | | | |
| --- | --- | --- | --- | --- | --- |
|  | Average test result  *Mean ±SD* | ICC_2,1_ (95%CI) | SEM | SEM% | MDC_95_ |
| External rotation | 101 N ±26 | 0.91 (0.81-0.96) | 11.7 N | 11.5 % | 32.4 N |
| Abduction | 108 N ±35 | 0.93 (0.84-0.97) | 11.2 N | 10.4 % | 31.0 N |
| Horizontal Extension | 127 N ±38 | 0.79 (0.45-0.91) | 53.4 N | 41.9% | 148 N |
| Protraction | 314 N ±115 | 0.92 (0.80-0.97) | 78.2 N | 24.9 % | 216 N |
